# A Quest for Mechanisms of Plant Root Exudation Brings New Results and Models, 300 Years after Hales

**DOI:** 10.3390/plants10010038

**Published:** 2020-12-25

**Authors:** Vadim Volkov, Heiner Schwenke

**Affiliations:** 1 Department of Plant Sciences, College of Agricultural and Environmental Sciences, University of California, Davis, CA 95616, USA; 2 K.A. Timiriazev Institute of Plant Physiology RAS, 35 Botanicheskaya St., Moscow 127276, Russia; 3 Max Planck Institute for the History of Science, Boltzmannstraße 22, 14195 Berlin, Germany

**Keywords:** root pressure, exudation, xylem embolism, mechanosensitive ion channels, ion transporters, aquaporins, water transport

## Abstract

The review summarizes some of our current knowledge on the phenomenon of exudation from the cut surface of detached roots with emphasis on results that were mostly established over the last fifty years. The phenomenon is quantitatively documented in the 18th century (by Hales in 1727). By the 19th century, theories mainly ascribed exudation to the secretion of living root cells. The 20th century favored the osmometer model of root exudation. Nevertheless, growing insights into the mechanisms of water transport and new or rediscovered observations stimulated the quest for a more adequate exudation model. The historical overview shows how understanding of exudation changed with time following experimental opportunities and novel ideas from different areas of knowledge. Later theories included cytoskeleton-dependent micro-pulsations of turgor in root cells to explain the observed water exudation. Recent progress in experimental biomedicine led to detailed study of channels and transporters for ion transport via cellular membranes and to the discovery of aquaporins. These universal molecular entities have been incorporated to the more complex models of water transport via plant roots. A new set of ideas and explanations was based on cellular osmoregulation by mechanosensitive ion channels. Thermodynamic calculations predicted the possibility of water transport against osmotic forces based on co-transport of water with ions via cation-chloride cotransporters. Recent observations of rhizodermis exudation, exudation of roots without an external aqueous medium, segments cut from roots, pulses of exudation, a phase shifting of water uptake and exudation, and of effects of physiologically active compounds (like ion channel blockers, metabolic agents, and cytoskeletal agents) will likely refine our understanding of the phenomenon. So far, it seems that more than one mechanism is responsible for root pressure and root exudation, processes which are important for refilling of embolized xylem vessels. However, recent advances in ion and water transport research at the molecular level suggest potential future directions to understanding of root exudation and new models awaiting experimental testing.

## 1. Introduction

**Figure 1:**
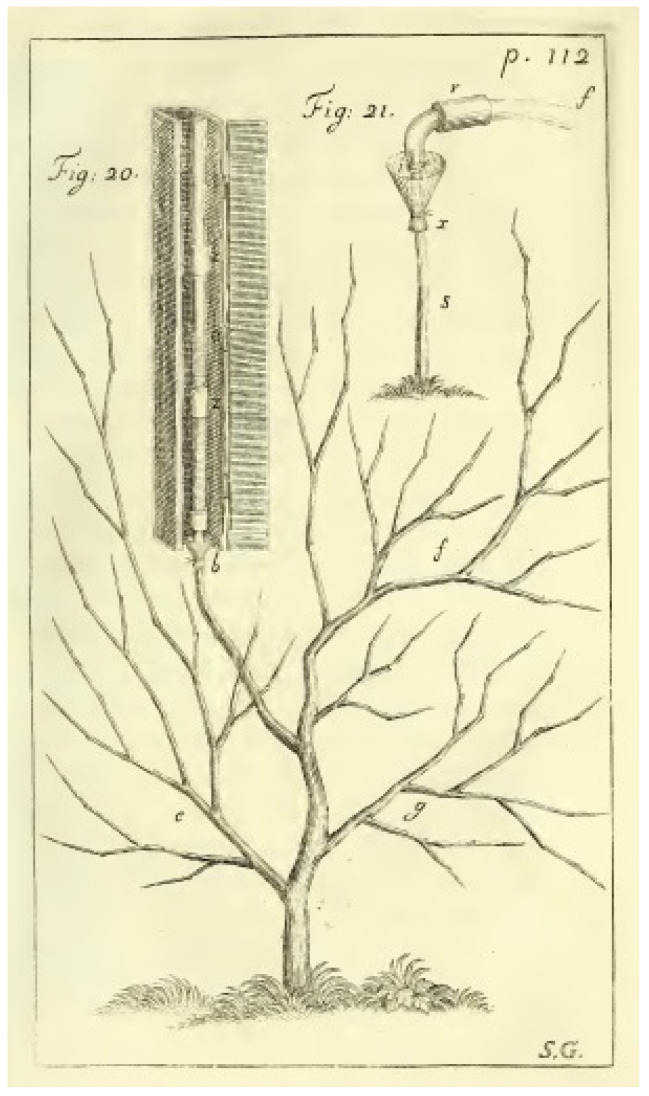
A sketch illustrating experiments done by Hales before 1727 [1] where mercury manometers were tightly connected to the cut branches of leafless grapevines to measure the pressure of the sap extruded by roots (reproduced from [1] with the permission based on https://library.si.edu/digital-library/book/vegetablestatick00hale).

Roots of many plant species show extrusion of liquid from their cut surface, typically when located in wet soil or submerged in distilled water or a solution. The observation of root exudation goes at least back to Theophrastus (*Historia plantarum* 9,1,6). Albertus Magnus tried to explain it by a *spiritus pulsatilis* (*De vegetabilibus* 2,1,3; see also 6,1,15). About 300 years ago, Hales seems to be the first who did quantitative measurements of the pressure that arises from exudation [1]. He attached mercury manometers to cut roots and branches of typically 3–5-year-old grapevines (Figure 1). Hales found that the observed pressure developing by the extruded sap was around 0.1 MPa (1 bar or approximately 10 m of water column) in spring months for leafless plants. Results on the prevalence of root exudation vary. Clark, for example, reported in 1874 that he tested “more than sixty species of trees and shrubs …, by boring a three-quarter inch hole usually to the depth of two inches into the sap-wood near the earth”, and apparently found that only a minority “showed any tendency to bleed” ([2], pp. 26–27). Recently, a study of tropical vines and woody plants found that only 61 of 109 species exhibited predawn root exudation from shoots or lateral branches [3]. On the contrary, 19th century researcher Kraus, who focused on the youngest parts of the roots, observed root exudation in all of the almost hundred herbaceous and seventeen woody species he examined: among them were seven coniferous species [4]. He therefore considered it highly probable that all younger root parts can exude from cross sections. In 1893, Wieler who listed 188 species from 65 families showing exudation after cutting all or a part of the epigeal parts, came to a similar conclusion [5]. However, it obviously depends very much on the method and the physiological status of the root, whether exudation can be observed or not. For example, in conifers, root exudation could be stimulated by long-term cold storage [6], while earlier any exudation was rarely observed in situ for whole conifer root stocks [7].

Most experiments on root exudation and root pressure were conducted with herbaceous plants (tomato, maize, cotton, castor beans, kidney beans, sunflower, barley, onion etc.), which are often simpler for experimental procedures and faster to grow than woody species (a short non-comprehensive representative list of references (mostly arranged by years) includes: [8,9,10,11,12,13,14,15,16,17,18,19,20,21,22,23,24,25,26,27,28,29,30,31,32,33,34,35,36,37].

## 2. Root Pressure and the Ascent of Sap

Although many investigations only determined root pressures in the range from slightly above zero to 1.5 bar (see [5], p. 112, for various woody and herbaceous species; [18]: 1.2–1.4 bars for maize; [38]: 0.5–1.5 bars for oak; [3]: 0.02 to 1.48 bars for 61 tropical vines and woody species; and [39]: 0.01–0.08 bars as estimates for two submerged aquatic angiosperms, *Lobelia dortmanna* and *Sparganium emersum*), higher pressures have been occasionally reported. Wieler measured more than 1.8 bar in *Betula alba* ([5], p. 123) and Clark 2.5 bar in *Betula lenta* ([2], p. 33). Values from 1.3 to 4.0 bars were registered for barley roots [37]. For detached roots of *Zea mays*, [17] reported root pressure values around four bars. The maximal measured and reported values of root pressure were above six bars for tomato roots [8,9].

Such values suggest that water might be raised to heights below 10–40 m. However, simple comparison with water potentials (Ψ_w_) driving transpiration under low humidity shows that root pressure is relatively low: change in air humidity from 100% to 99% results in decrease of Ψ_w_ by 1.38 MPa (R × T/V × 0.99, about 13.8 bars) where R is the universal gas constant equal to 8.31 J/(K × mole), T is temperature in K, V is the partial molar volume of water equal to 18 cm^3^. Hence, the root pressure might be important under low transpiration for leafless non-transpiring plants or in a highly saturated humidity.

The importance of root pressure and exudation can be considered from an integrative point of view when the water flow transfers ions, metabolites, and physiologically active compounds linking all parts of the plants. Root pressure unites the parts of a multicellular organism when large transport fluxes are carried by directed water fluxes. This revives the idea of circulatory system of plants which was—apparently independently—developed between 1665–1668 by Johann Daniel Major, Christopher Merret, Claude Persault, and Edme Mariotte ([40], p. 1); for recent proponents see [25,41,42] and references therein). From the more common point of view, root pressure is important for refilling the cavitated/embolized xylem vessels in the spring time and under low transpiration (e.g., [43]).

Embolism of xylem vessels (filling with air with consequent loss of conductivity for xylem sap) occurs under water stress, winter freezing, or sometimes after attacks of pathogens [43,44]. The process may result in wilting and further death of plants: if all the conductive elements are embolized then water cannot be transported to shoots. Hence, root pressure is important for refilling the xylem vessels for small trees and vines. For wild grapevines over winter, the xylem vessels are filled with air with usually no sap inside; in spring before the leaf expansion the root pressure in some of the vines reaches up to five bars filling the xylem vessels [43]. Measurements for 15 vines in spring registered root pressure up to twofold above the required pressure based on the height of the vines (up to 30 m); for transpiring vines in summer the root pressure was quite low if any. The experiments also revealed that the water path might be more complex in vines with (partially) embolized xylem and, interestingly, higher root pressure corresponded to taller vines [43]. Refilling of xylem by root pressure in grapevines requires either dry pit structures between vessels with expulsion of air or more complex mechanisms [44,45] when air is dissolved within individual vessels separated by hydrated pit fields. Evidently, this is applicable to other plants. The pit structures/membranes connecting xylem vessels have narrow pores of submicrometer to-nanometer range, e.g., with maximal diameters of 39 nm in *Vitis vinifera* and 225 nm in *Betula pendula*: [46] (reviewed in: [47]).

Wet pit membranes seal individual xylem vessels with pressures typically over 1.5–3 MPa (15–30 bars) in accordance with the simplified Young–Laplace equation:

ΔP = 2 *γ*/*r* (1)

where ΔP is access pressure formed, *γ* is surface tension of water (about 72 mN/m with slight dependence on temperature), and *r* is the radius of the pore. A new tensiometer with a nanoporous membrane obeying the Young–Laplace equation was designed recently [48] and is being used in trials to determine stem water potential in grapevines, apple trees, almond [49]; it cavitates below −100 bars. The same equation can be applied to the air in wet embolized xylem vessels. The air is pressurized by thin water film adjoining the surface of the vessels, the extra pressure for xylem vessel with diameter 10 μm is about 0.3 bars (30 kPa). Hence, to dissolve the air in the xylem vessel with radius *r* an extra pressure over 2 γ/*r* is required. It is produced mostly by root pressure in grapevines [43,45], maize [50], many tropical plants [3], 53 of 53 studied bamboo species [51]. When root pressure is not generated or not sufficient for xylem refilling, the more complicated mechanisms of xylem refilling at the expenses of water and ion transport of xylem parenchyma cells are involved [52,53,54,55]; the mechanisms of which are not discussed here in detail, although their nature could be similar to that of water transport in roots (see about osmotic and nonosmotic components of root pressure below). Obviously, the mechanisms may operate for refilling the xylem conduits in tall trees such as sequoia (when possible) where the gravitational part of water potential Ψ_w_ exceeds −1 MPa (−10 bars), even leading to symptoms of water deficit for high leaves [56].

One more expression of root pressure is guttation or release of water by plants from their leaves, often via specialised structures named hydathodes. Guttation occurs less frequently than root pressure and exudation: for 109 tropical vines and woody plants, 61 of 109 species exhibited predawn exudation while predawn guttation was recorded for only 15 [3]; guttation was reported for 8 of 53 bamboo species with detected root pressure [51]. Moreover, guttation did not always correspond to high root pressure. Species with maximal root pressure from 0.16 to 1.2 bars had guttation; root pressure for the set of measured species varied from 0.02 to 1.5 bars [3]. For guttating bamboo species, the osmotic pressures of the extruded liquid were often over 10 times higher than the recorded by manometer root pressures; the researchers suggested low reflection coefficients (see below) for the coupling of water and ion fluxes [51]. More details about guttation and its implications for the life of plants are reviewed in [57,58,59] with extensive description and references therein).

## 3. Origin of Exudate

Rowan, McCully, and Canny [35] investigated the origin of the root exudate on cut surfaces of detached maize roots, root segments and cortical sleeves by means of optical microscopy and cryomicroscopy. After preparation of a specimen, they waited until a dome of exudate had formed on the cut surface, blotted it and observed the appearance of further fluid over the various types of tissue during the seconds following the blotting. They found that the exudate from the cut surface came from all cell types. It appeared from the cortex, epidermis, and hypodermis first, and only later from the xylem vessels, pith parenchyma, and aerenchyma spaces [35]. These results confirmed and added to a number of early investigations that were carried out by means of a hand lens by Dutrochet ([60], p. 13; [61], p. 369), Rominger [62], Brücke [63], Kraus [64], and James and Baker [65]. By far the most comprehensive study of this kind was carried out by Kraus ([64]; see for his method [4], pp. 19–20). After studying numerous herbaceous and woody plants, he concluded that the exudate originated from living cells of various types of tissue ([64], pp. 2–9, 31). James and Baker, who examined the exudation of sycamore roots and stems with a hand lens on cut surfaces and also with live stains, found that the primary source of exudate was living tissue and not the vessels, opposite to most earlier observations with other plants [65].

However, xylem elements are definitely the main pathway for ascending water flow in plants as confirmed by magnetic resonance imaging (MRI) experiments for 6-d-old castor bean seedlings (*Ricinus communis*) [66]. The measured rate of flow in xylem of intact plants under low transpiration was similar to exudation rates measured for detached roots of castor bean seedlings of the same age [67]. MRI experiments also demonstrated large recirculation of water between xylem and phloem and similar flow rates (Stokes flow with rates around 2 m/h) for the two specialised tissues while larger cross-section xylem area ensured higher total fluxes in the xylem [66]. MRI experiments demonstrated higher xylem flow under transpiration with precise spatial resolution of individual large vessels and refilling of cavitated xylem vessels in cucumber [68]. The results of MRI for plant water transport in xylem and phloem are widely reviewed (see, e.g., [69,70] and references therein). So, the large water exudation flows via detached roots, such as 11,260 mm^3^ exudation fluid from a decapitated *Urtica urens* (annual nettle) over 2.5 days, although the volume of the root was only 1450 mm^3^ [71], are to be via specialised water-conducting xylem tissues with high hydraulic conductivity.

Another approach to determine the origin of the exudate was to analyze its chemical composition. According to pH, ion composition, contents of amino acids and soluble sugars, the exudate of detached roots of maize seedling comes mainly from the xylem, not from the phloem (Table 1, based on calculations of osmotic pressure, see the next chapter, ions and amino acids make up 90% of osmotic pressure of this exudate with sugars below 5%) [23,24]. Amino acid analysis demonstrated that Asn and Gln were the main free amino acids of exudate making up to 40% of all free amino acids; these were followed by Val and Ser (20% more) [24]. Phloem exudates are more alkaline (pH over 7.2–7.5) with much higher osmotic pressure (over 8 bars) and different chemical composition [72,73,74] and references therein.

A xylem origin of exudate was evident in other experiments with maize [17], castor beans [75], tomato [76], tobacco [77], poplar [78], kiwifruit vines [79], grapevines [80,81], and even in the much earlier experiments with maize and *Impatiens balsamina* [82]. Similar results were obtained for conifers [83]. (However, chemical composition of exudates exhibited certain variability depending on the time of the day and even the month of exudate collection for field-grown plants [76,78,80].)

## 4. Initial Concepts and Further Models for Understanding Mechanisms of Root Pressure and Exudation

Hales explained the rising of the sap in the plants with the “strong attraction of the capillary sap vessels [...] assisted by the brisk undulations and vibrations, caused by the sun’s warmth” ([1], p.136). After Nollet discovered osmosis in 1748 [84], it took almost eighty years before Dutrochet attempted to explain root exudation in osmotic terms [60]. Like Hofmeister and Sachs later on, he saw the main cause of this phenomenon in the water expulsion from turgescent root cells ([60], p. 162; [85], p. 175; [86], p. 204). Pressure secretion from living cells was the leading exudation concept of the 19th century (see [5] for the most comprehensive overview of 19th century exudation research). However, only Pfeffer was able to develop physically correct models of root exudation. He built the first functioning osmometer, the so-called Pfeffer’s cell [87]. Based on his measurements, van’t Hoff formulated the law named after him [88], which is in its simplest version

π = c × R × T(2)

where π is the osmotic pressure, c is the concentration of the solutes, R is the universal gas constant (8.31 J/(K × mole)), and T is the temperature in Kelvin. Rough estimates show that 40 mM of solute which does not dissociate (or 20 mM for completely dissociating salt XY producing monovalent ions X^+^ and Y^−^ etc.) make up 1 bar (about 0.1 MPa) of osmotic pressure.

Pfeffer basically considered two variants of exudation by pressure secretion. According to the first, the permeability of plasma membrane to dissolved substances would be different on opposite sides of a cell. Today, we would say that the reflection coefficient of the plasma membrane varies at different sides of the cell. This would allow the cell to absorb water on one side while releasing it from another. The second model employs an intracellular gradient of solutes. Thus, a pressure secretion of water on the side of the lower osmotic pressure can be achieved. Pfeffer also considered what he called passive or plasmolytic secretion. In this case, the water is sucked from the cell by dissolved substances outside the plasma membrane. This would be the basic idea of the osmometer model of root exudation, which became popular later. Pfeffer considers this mechanism suitable for exudation from nectaries, but not the main cause of root exudation. He found the exudate often lacking significant amounts of dissolved substances and stated that exudation pressure was not correlated with the osmotic concentration of the exudate. Moreover, his co-worker Wieler could not obtain any exudation in inactive plants when he filled the glass tube, which was attached to decapitated stumps, with 0.1–1 percent solution of potassium nitrate. In any case, Pfeffer regarded exudation as a consequence of energy-consuming activity of living cells, referring to experiments by Wieler on the effect of chloroform and oxygen deprivation on exudation ([5]; [87], pp. 224–234; [89], pp. 299–319; [90], pp. 265–267; [91], pp. 234–268).

Since the publications of Atkins [92], Priestley [93] and Sabinin [82] the osmometer model of root exudation has been increasingly favored. The driving force for root exudation is assigned exclusively to the difference in osmotic pressure between the xylem and the external medium, although studies from the 19th century had already shown that this explanation does not fit the data well. Nevertheless, the osmometer model became the leading exudation model in the 20th century (see, e.g., [94,95]). Its main appeal may have been that it offered a simple mathematical formula for the driving force behind root exudation.

Further development of exudation studies in the 20th century productively absorbed Onsager’s framework of irreversible processes (reviewed in: [96] and the references therein). Instead of the earlier simple Equation (2), Kedem and Katchalsky applied Onsager’s theorems on linear irreversible thermodymanics from membrane processes [97] to fluxes of ions and water via membranes [98,99,100]. The approach introduced equations for water (for living systems or solvent in general) and ion/solute fluxes with several additional coefficients:

J_w_ = −L_p_ × (ΔP − σ × ΔC_s_ × R × T) (3)

**Figure 2:**
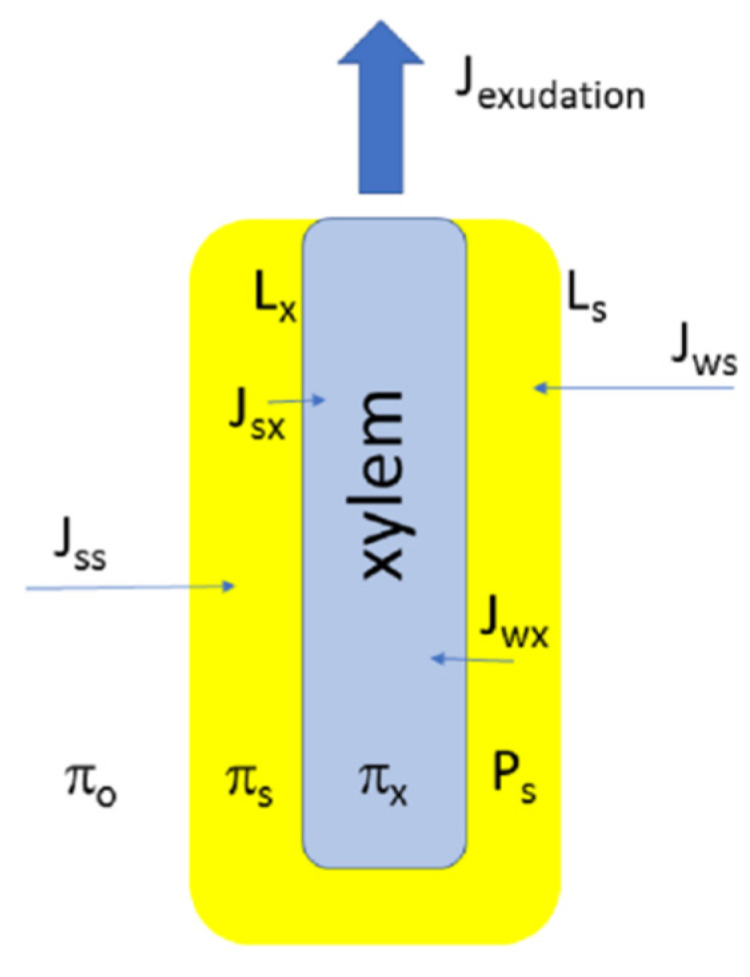
Basic scheme of root often considered for modelling exudation flow J_exudation_. L_s_ and L_x_ are hydraulic conductivities of symplast and at the boundary symplast-xylem, correspondingly. The fluxes of solutes (mainly ions) are symbolized by s (J_ss_, J_sx_), the flow of water by w (J_ws_, J_wx_), the second subscript (s or x) indicates the layer (s for symplast) or boundary (x for symplast-xylem, correspondingly). The outer osmotic pressure is taken as π_o_, the osmotic pressure of xylem is π_x_. More complex models add osmotic π_s_ and turgor P_s_ pressures of symplast; then specific reflection coefficients σ are introduced for the models for different compartments which show the coupling between water and ion fluxes. The relations between water J_w_ and solute J_s_ fluxes are based on Equations (3) and (4) of linear irreversible thermodynamics. The sketch is a generalized scheme since each model usually has numerous specific features.

J_s_ = −ω × ΔC_s_ + (1 − σ) ×C_s_ × J_w_ (4)

where J_w_ is water (or solvent) flux across the membrane, L_p_ is hydraulic conductivity of membrane, ΔP corresponds to difference of hydrostatic pressures across the membrane, the second term of (3) corresponds to difference of osmotic pressures across the membrane multiplied by the reflection coefficient σ J_s_ is solute flux, ω is solute permeability, and C_s_ is similar to averaged solute concentration for compartments (more precise definitions of the parameters require more complex explanation and could be found in the cited papers). The equations were swiftly and productively applied to plant water relations (commencing from Dainty [101]) and then to models of root exudation (Figure 2; Table 2). For water fluxes, the reflection coefficient σ varies from 0 to 1 and determines how osmotic forces are translated into the driving forces for water transport. The reflection coefficient σ is a measure of leakiness of biomembranes for solutes (σ equals 1 for a membrane completely non-permeable for solutes).

We briefly describe the models of root exudation (Figure 2; Table 2) which are mainly based on an osmotic explanation of exudation alone, starting from the earlier one membrane models with two compartments (with all the root tissues assumed as one membrane between compartments of external medium and root xylem) to more complicated two membrane models with three compartments (compartments of external medium—root tissues—xylem, each has specific ion concentrations and similar or different reflection coefficients for fluxes between external medium—root tissues, root tissues—xylem).

Models of root exudation with a simple osmotic explanation and one important membrane stem from Atkins [92], Priestley [93], and Sabinin [82], followed later by, for example, Eaton [94]. Calculations (where provided) from the investigators demonstrated a linear relation between exudation rate and external osmotic pressure (or even between exudation rate and the difference of exudate and external osmotic pressures) confirmed in experiments. Further approach from linear thermodynamics added the reflection coefficient σ and more complexity. The simple osmometer models of exudation could be partially amended (when failing to explain new experiments, e.g., by van Overbeek [102] etc.) by using variable reflection coefficients and variable hydraulic conductivities for the membranes involved. However, more complex models gained more attention and looked promising (see below). Again, the models mostly operated at the level of a whole root, averaging tissues, and summing up their properties with several membranes having specific reflection coefficients and determined hydraulic conductivities with associated water and solute fluxes. The membranes and compartments are defined with the parameters of fluxes, reflection coefficients and also turgor pressure of cells (for root symplast) (Figure 2; Table 2). These models of exudations include that of (1) Anderson, Aikman and Meiri [103] who proposed a concentration gradient within the vessel lumens; (2) Ginsburg [104] for iso-osmotic flow; (3) Katou, Taura and Furumoto [105] with standing gradient flow; (4) Lyalin [106] with a reverse-osmosis model and (5) the recent model of Pickard [36,107], precisely describing numerous introduced parameters for water and ion transport via roots. Most models and their limitations are well reviewed in [22] (where another classification of the models is introduced): the models can explain potential exudation against reverse osmotic gradients but still many aspects of root exudation are not predicted by the models. For example, the predicted standing gradient of osmotic pressure in xylem vessels [103] was not revealed for maize roots [108]. In contrast to the predictions of the osmometer models, osmotic pressure in the same xylem vessels was relatively stable under changing osmotic pressure of external solution while osmoregulation occurred in the vacuoles of the living root cells [109]. Exudation of roots exhibited sharp pulses within tens of seconds, responded to a wide set of selective inhibitors and metabolic regulators (see below).

**Table 2:** Main types of quantitative models for describing the phenomenon of root exudation ×.

Type of Model, Main Feature	Short Description	Principle	Proponents, References
Macroscopic	Simple osmotic idea that exudation is caused purely by difference of osmotic pressures between xylem sap and external medium; exudation rate is linearly proportional to this difference	osmotic	Atkins [92], Priestley [93], Sabinin [82], Eaton [94] and others
Macroscopic	Concentration gradient within the vessel lumens drives water flow and exudation	osmotic, standing gradient flow	Anderson, Aikman and Meiri [103]
Macroscopic	Iso-osmotic flow is realized due to different parameters and reflection coefficients for membranes located in a row	osmotic, iso-osmotic flow	Ginsburg [104]
Macroscopic	Concentration gradient exists within the cell walls inside the root stele; water follows osmotic forces which are caused by solute transport, potentially at the expenses of energy for ion fluxes	osmotic, standing gradient flow	Katou, Taura and Furumoto [105]
Macroscopic	High turgor pressure in the root symplast allows reverse osmosis and nonlinear dependence between exudation rate and difference in osmotic pressures between exudate and external medium	reverse osmosis at the level of whole root symplast	Lyalin [106]
Macroscopic	Detailed model with over tens of coefficients, microscopic coefficients are based on structure of plasmalemma and applied to averaged membrane interface between symplast and xylem; assumes active ion fluxes	models osmotic effects with reference to the microscopic level without irreversible thermodynamics	Pickard [36,107]
Subcellular	Reverse osmosis is realized in each plasmodesma of root endodermal cells; turgor oscillations within the cells at 1 Hz frequency result in pumping of water to the root xylem, produce uphill water flux and exudation; energy is pumped into the system via mechanic constrictions and cytoskeleton	reverse osmosis at cellular and subcellular levels	Kundt, Robnik [110,111]
Molecular	The first known model introducing specific ion channels to water transport; alterations in reflection coefficient due to mechanosensitive ion channels explain oscillations in exudation; combination with reverse osmosis may explain visible non-osmotic effects	alterations in reflection coefficient between root compartments with potential implications of reverse osmosis	Schwenke [21], Schwenke, Wagner [22]
Molecular	Model of active water transport based on water cotransport with cation-chloride cotransporters, transferred to plants from animal physiology; suggests the mechanism of water transport to xylem vessels; applicable to root exudation	energetically uphill water transport due to water molecules cotransport with cation-chloride cotransporters	Wegner [112,113,114]; Fricke [115]

× Due to the large number of models, the Authors are not able to include all of them here; more models and details are given in [22]. Moreover, many models do not provide exact quantitative description, so it is difficult to classify them within the frames we have used.

Here we pay more attention to the recent models that provide links to the cellular details of root structure and to molecular entities for ion and water transport. Over the last decades models of exudation and description of water fluxes in plants gained a lot from molecular studies when ions channels, transporters and specialised proteins, aquaporins for facilitated water flow, were discovered. The model with periodically changing reflection coefficients [21,22] was the first (in our knowledge) where macroscopic properties were linked to the known molecular dynamics of individual mechanosensitive ion channels. Our knowledge of mechanosensitive ion channels of plants is still insufficient (see below) to identify individual ion channels to confirm or amend the model.

The other ion transporting proteins suggested to participate in root water transport are cation chloride cotransporters [112,113,114,115]. The cotransporters of cations and glucose proved to additionally transfer hundreds of water molecules per transported substrate in animal systems and can be put under heterologous expression in *Xenopus* oocytes [116,117,118,119]. The existence of a similar mechanism in plants was explored thermodynamically and considered feasible for water transport to xylem vessels [112,113,114,115]. Again, we await experimental confirmation of the hypothesis for plants.

The discovery of aquaporins posed questions of their importance to the basics of water transport in plants (see, e.g., [120]); their role in water transport via roots was demonstrated (see, e.g., [121,122]) and developed into a wide, intensively growing area for research. Questions of whether the discovery requires modification of basic equations of linear thermodynamics for water and solute fluxes still remain (see, e.g., [120]). The membrane reflection coefficient generated by many individual aquaporins depends on the nature of solutes at the sites of the aquaporins (these aquaporins are permeable to low molecular weight uncharged molecules); hence, knowledge of the solutes generating the osmotic gradients is required. Aquaporins can be concentrated in specific areas within membranes generating high hydraulic conductivity and so contribute to the formation of mosaic composite membranes at different spatial scales (as suggested by, e.g., [123]). Anyway, the evident effect of aquaporins on root hydraulic conductivity L_p_ has been demonstrated in most conducted experiments (from 0% up to 90% of L_p_ is linked to aquaporins as summarized in [122]).

## 5. Rhizodermis Exudation

**Figure 3:**
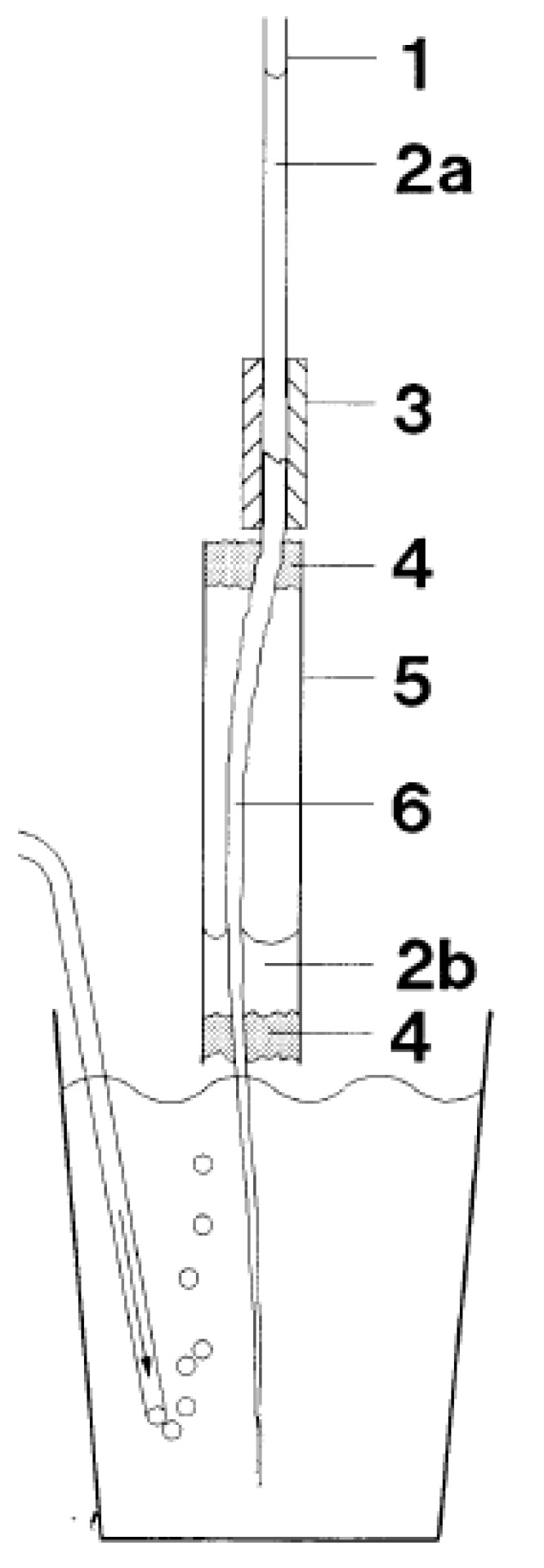
Experimental setup for demonstrating rhizodermis exudation: (1) capillary; (2a) xylem exudation; (2b) rhizodermis exudation; (3) rubber joint; (4) silicon rubber cement; (5) glass cylinder; and (6) maize root. The lower part of the root was immersed in aerated distilled water at constant temperature of 23 °C. The experiments lasted 2.5 days, rhizodermis exudation occurred in all 25 maize roots tested. Reproduced from [22] with the permission of the publisher John Wiley and Sons.

The phenomenon of root exudation presumably requires polarity in water flows. The axial, and to a lesser extent radial, polarity is ensured by root morphology and the direction of transport processes by the distribution of transporters, uneven osmotic pressures of liquids and even by measured polarity in hydraulic conductivity for water flows (see, e.g., [18] for conductivity measurements). Since water is usually absorbed by roots, the observation that roots of maize seedlings exuded liquid radially to the outside of the roots over days, simultaneously to xylem exudations ([22]; see below), was unexpected. The middle part of roots was encased and exuded water to a closed water-free compartment over several days at constant temperature (Figure 3). At the same time exudation into micropipettes attached to the cut end of the root was observed [21,22]. These phenomena were reproduced in another study [124] using similar equipment. Rhizodermis and xylem exudation were absent in maize roots boiled for 5 min in tap water [124]. Water condensation as the explanation of the phenomenon was rejected [22,124]. Moreover, 11 woody species (*Abies alba*, *Acer pseudoplatanus*, *Alnus glutinosa*, *Betula nigra*, *Carpinus betulus*, *Fagus sylvatica*, *Fraxinus excelsior*, *Prunus avium*, *Quercus robur*, *Quercus rubra*, and *Vitis vinifera*) were examined. All of the species showed concomitant rhizodermis and xylem exudation, except *Carpinus betulus* where only the cut surface became moist [124].

Similar results were obtained by a different experimental approach: efflux of water from the surface of field-grown roots of maize, oats, barley, and crabgrass was confirmed by means of cryo-scanning electron microscopy. This efflux from the root surface coincided with guttation from leaves. From the convex shape of the droplets it was concluded that water was not drawn passively from the root by lower potential of the soil. Excavated and subsequently frozen roots of the plants were analyzed, at the point where ice crystals on their surface evidenced the extrusion of water to the soil. These crystallized droplets of water were abundant in the early morning; none or very few were observed for roots collected at midday. The chemical X-ray analysis indicated the presence of carbon, potassium, phosphorus, and calcium in the regions of the droplets [125].

There had been much earlier reports of water efflux from the surface of roots, mostly from root hairs. According to De Candolle ([126], p. 248), Brugmans was the first to observe small droplets oozing from the tips of the rootlets of *Viola arvensis* at night. In 1888, Molisch [127] reported that he quite often observed liquid excretions of roots, similar to guttation from leaves. He judged the droplets were not dew because they appeared only at the tip of healthy roots hairs, on healthy root hairs only, and they would also appear when the roots were kept in a room whose temperature was gradually increased so that no condensation of water would occur. In the same year, Stahl ([128], p. 42) reported drop formation on root hairs of *Circaea lutetiana* observed under a microscope. Czapek [129] found it easy to demonstrate liquid root excretions. If young seedlings of grasses were grown in steam-saturated rooms, as soon as an abundant development of root hairs has occurred, fine, colorless droplets would be seen on many of the latter. These droplets would appear only when the hair cells were highly turgid. Pfeffer ([91], p. 257) wrote that water droplets are generally excreted by root hairs in a saturated atmosphere and considers intracellular excretion under pressure to be the probable cause. When there is bleeding pressure (from the xylem of a root), some of the otherwise absorbing parts of the root would excrete water. Thereby, he seemed to postulate the simultaneity of xylem exudation and rhizodermis exudation. In the 20th century, Rogers [130] documented by microphotography copious droplet formation on the surface of apple roots in the regions with older root hairs. Richardson [131] observed droplets on root hairs of *Acer pseudoplatanus* but ascribed them without further experimental scrutiny to condensation. Cailloux [132,133,134,135] measured exudation from oat root hairs and normal rhizodermal cells at high resolution by means of a micro-potometer. Head [136] filmed droplet development on the hairs of apple roots by time-lapse cine-photomicrography.

For detached roots of 40–60 days old sunflower plants, which were removed from water and their surfaces dried, exudation continued for days if the roots were contained in dry sealed glass flasks. At the same time, some water also accumulated at the bottom of the flasks: this water was explained by evaporation from external root surface. Howeverthe possibility of rhizodermis exudation was not widely known or discussed at the time [27,29]. For one of the experiments, a root lost, on the average, 11 g of water within two days (from 65 g to 54 g), the weight of root exudate was 5 g while 6 g were collected from the bottom of the vessel containing the root [29].

Rhizodermis exudation could help explaining the unexpected observation of increasing soil water contents towards the root surface, measured by neutron tomography, in chickpea, white lupin, and maize ([137], for further discussion see [138]). It seems likely that rhizodermis exudation contributes to the phenomenon of hydraulic redistribution. Hydraulic redistribution is the transport of water from moist to dry soil zones via the root system during periods of low transpiration [139,140,141]. It can take place upward (hydraulic lift) [142,143], downward (inverse hydraulic lift) [144,145] (see earlier: [146,147]) and laterally [148]. Through hydraulic redistribution, plants can create a water buffer outside the roots and improve the availability of nutrients [138,149,150]. Because hydraulic redistribution allows for water parasitism, it can be used for a natural irrigation system in agriculture through the combined cultivation of shallow-rooted crops with deep-rooted plants capable of a pronounced hydraulic lift [151,152,153].

The efflux of water and organic matter with ions from the roots to rhizosphere is important for interactions of plants with soil microorganisms, soil insects and nematodes and with the other plants. The subject is intensively studied nowadays with fruitful practical and theoretical implications for plant life and stress tolerance (see, e.g., for questions posed earlier and references in: [10], recent reviews and research papers include: [154,155,156,157,158]. Roots release a wide spectrum of metabolites ranging from amino acids and sugars to alcohols, organic acids, nucleic bases and nucleotides, secondary metabolites such as alkaloids, phenylpropanoids, acylsugars, and terpenes (see, e.g., [157,158,159] with references therein). The concentrations and proportions of the metabolites released provide specific biochemical signatures of growing roots along their length [157], presumably function in root-to-root signalling [159] and serve as a competitive trait of fine roots under field conditions [160]. The profile of root-released metabolites changes under drought and influences rhizosphere-inhabiting microbes [161]. Similarly, the alleviating effect of certain microbial communities was shown under salinity stress for some plants (see, e.g., [156] on specific strain of *B. subtilis* for clover). Mechanisms of transport for the released metabolites involve specific ABC and MATE transporters and simple and facilitated diffusion [157]. However, it is very likely that the metabolites make up only a very small fraction of osmotic pressure in any root exudates (e.g., compare to Table 1). Therefore, understanding of the rhizodermal liquid exudation would help to predict how the concentrations of metabolites are regulated in rhizospheres. *Visa versa*, the study of genetic and molecular basis for release of metabolites to rhizospheres would provide more information to aid understanding of the phenomenon of rhizodermis exudation.

## 6. Exudation of Root Segments without Root Apex and Stem Exudation

**Figure 4:**
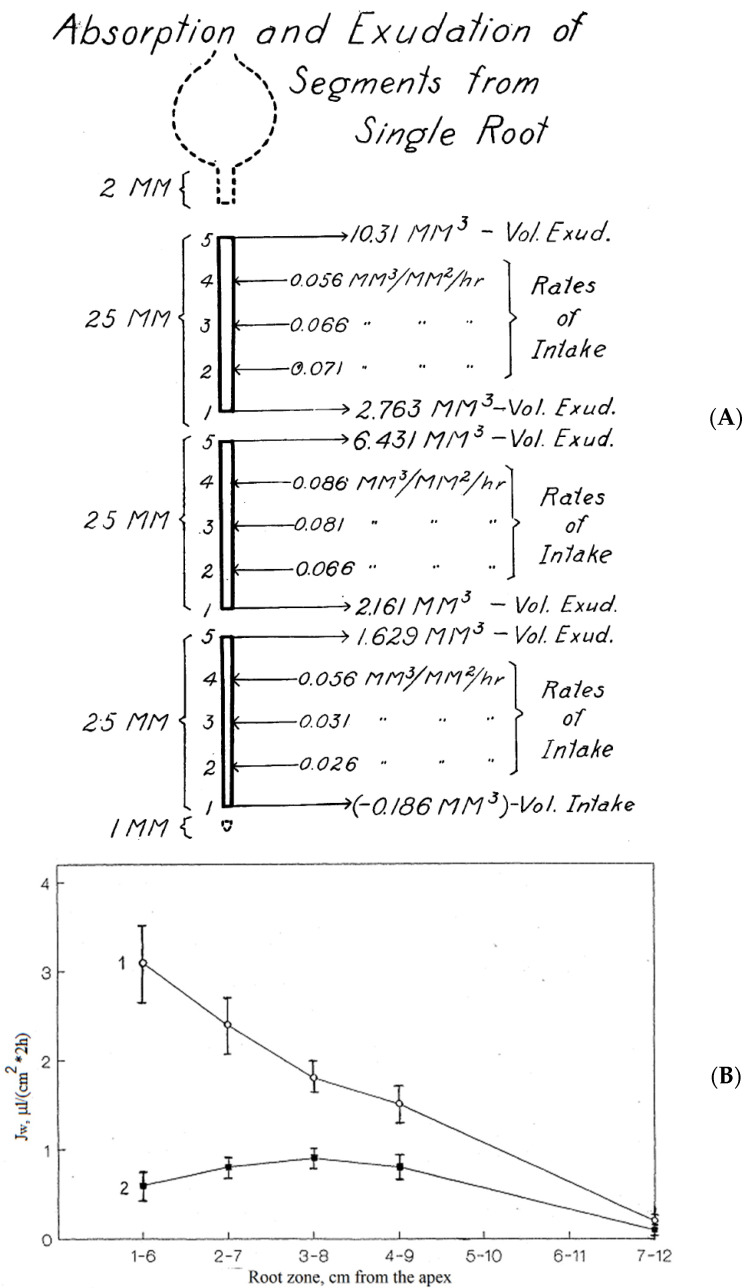
Exudation of root segments isolated from onion roots (**A**); reproduced from: [10] with the permission of American Society of Plant Biologists with further enhancement by AI software and roots of maize seedlings (**B**), reproduced from [26] equivalent to Figure 1 from [24]. A Diagram of total volume outflow and average rates of intake of water in isolated contiguous segments of the same root. Positions of the potometers are designated by the arrows numbered 1, 2, 3, 4, and 5. Observations were made during a 20-h period. B Exudation rate (J_w_) from basal (1) and the apical (2) ends of 5 cm root segments cut out from various root zones. Data are given as means ± SE. Exudation time was 2 h; the similar regularity was observed after the first 20 min of exudation and later on.

Effects of axial polarity and axial differentiation of roots arose in experiments with excised segments from roots. Exudation from root segments without a root apex and with xylem vessels cut at both ends was demonstrated for onion roots in a saturated atmosphere with several potometers attached along the roots [10,11]. The 25 mm long onion root segments absorbed water from three attached potometers along the axis and then exuded at the basal end (distal to the original apex), while the direction of fluid flow (uptake or efflux) at the apical end depended on the length of removed apical tissue (Figure 4A). Similarly, the discovery that root segments with vessels cut at both ends exude in both directions was later made independently for roots of maize seedlings in an attempt to check the single membrane osmotic concept of exudation [23,24,25,26].

Segments with the same length of 5 cm were cut from maize roots (the apical 2 cm were discarded, the next 5 cm of root were used in the experiments); these segments demonstrated exudation from basal end and also from the apical end [23,24,25,26]. The exudation rate from the basal end (distal to the original apex; direction for the exudation of detached roots) was usually 2–3 times higher than for exudation from the apical end (opposite direction to exudation of detached roots; 1.6 ± 1.2 μL/(h × cm^2^) compared to 0.7 ± 0.5 μL/(h × cm^2^), means ± SD; [25]). The chemical composition of exudates for segments from maize seedlings was similar to the exudate of detached roots, indicating xylem exudation, but exudate from the apical end (proximal to the original root apex) with slower exudation was more concentrated (II_i_ = 0.28 ± 0.03 MPa compared to 0.13 ± 0.03 MPa). The energy activation (expressed in Q_10_) for the faster exudation from the basal end was higher than from the apical end (see sections below for more explanations); exudation from both ends responded to metabolic inhibitors and stimulators and was stopped by the same external osmotic pressure from PEG 6000 (see sections below for more explanations) [23,24,25]. Exudation of maize root segments with the same length of 5 cm but cut at different distances from the root apex decreased as the segments were cut further from the apex. For exudation from the apical end (proximal to original apex) the highest exudation was for segments cut 3 cm from the original apex (Figure 4B). Sealing the apical end of maize root segments (2–7 cm from the root apex) with glue increased exudation from the basal end from 2.9 ± 0.3 μL/(h × cm^2^) to 4.7 ± 0.6 μL/(h × cm^2^) (means ± SE); sealing the basal end of maize root segments (2–7 cm from the root apex) increased exudation from the apical end from 0.5 ± 0.1 μL/(h × cm^2^) to 1.0 ± 0.1 μL/(h × cm^2^) (means ± SE) [26]. Presumably, the stage of development of xylem vessels along the roots was the reason for the differences in exudation from the ends of root segments. The phenomenon confirms the multiplex nature of root exudation and complexity of root structure. Interestingly, xylem-like (based on exudate chemical composition) exudation was recorded from the apical part of roots of maize seedlings exposed to air with 100% humidity when the apical 3–5 cm of the roots were cut off [162,163]; the researchers suggested that xylem pressure was generated in humid air and resulted in exudation after removing the hydraulically insulating root tip.

Still it is important to set the frameworks in generalizing our knowledge from several herbaceous plants to all plant species, especially for trees where measured xylem pressure could be caused by either by root pressure or by stem pressure. For example, xylem sap pressure in sugar maple *Acer saccharum* Marsh. is a basis for commercial production of maple syrup. The sap contains plenty of sugars (over 3%) and even flows under low temperatures from upright detached pieces of stem (trunks) and, moreover, from the reverted upside down stem pieces when they have their lower (morphologically apical) part in water [164]. The flow from maple stems exhibited large variability even from sections of one stem, dependent on temperature and required living tissues [165]. Likely, the mechanisms for the stem pressure and flow in some trees may differ from the mechanisms for root pressure in maize; in experiments with 17-years-old orchard walnuts both root and stem pressure were detected and separated based on temperature dependence and time of the year when they were dominant [166]. Xylem pressures up to 1.6 bars were recorded in autumn and spring, correlated with soil temperature, the pressures constituted about 55% of the calculated values based on xylem sap osmolarity and were attributed to root pressure. Lower xylem pressures up to 0.35 bars occurred in winter and were about 7% of the calculated values of sap osmolarity; these pressures were of stem origin [166]. So, several mechanisms could be in operation together for xylem exudation in different plants and their tissues.

## 7. Pulses and Oscillations in Exudation Rate, Coordinated Activity of Cells, and Estimates for Potential Changes of Cell Volume and Turgor Required

**Figure 5:**
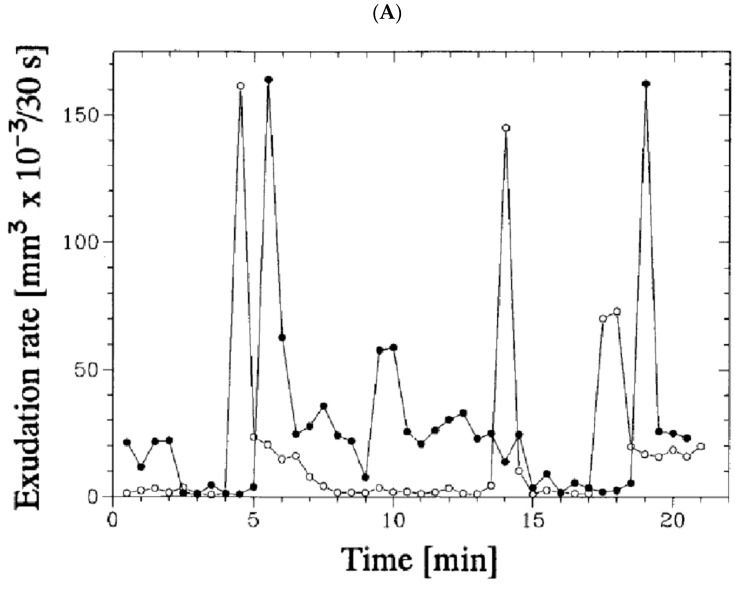

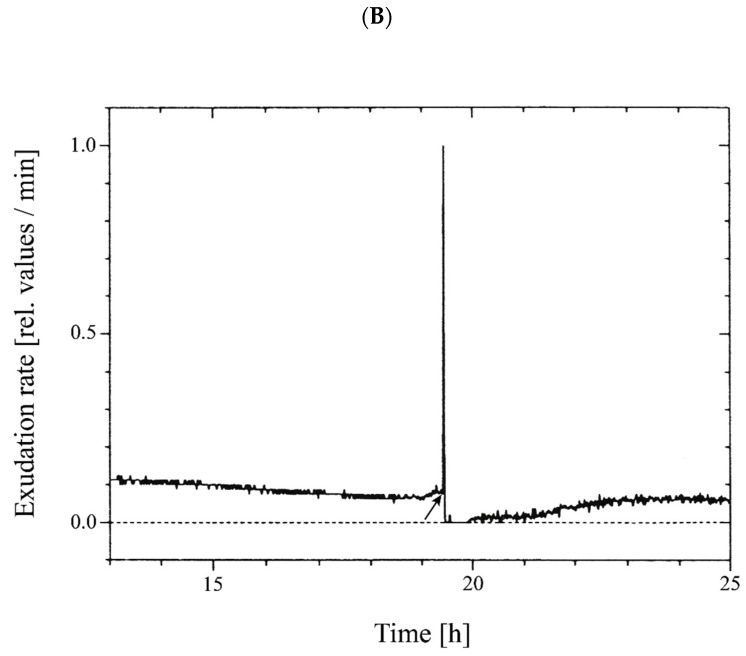
Exudation pulses recorded for detached roots of maize seedlings. (**A**) Rate of xylem exudation of two single maize roots in distilled water at constant temperature of 23 °C measured simultaneously by the micropipette method; standard error is 2.5 × 10^−3^ mm^3^. Reproduced from [22] with the permission of the publisher John Wiley and Sons. Similar results (though with lower temporal resolution) were obtained for detached maize roots of 5–7 days old seedlings at 30 °C [26]. (**B**) Collective exudation rate from the cut surface of 24 four-day old maize roots in aerated distilled water as bathing medium was measured by the micro drop recorder (see for this method [22]). The bathing medium was at a constant 22 °C up to the time indicated by the arrow. At this point, the temperature changed to 4 °C (cold bathing medium by pot change). The ensuing exudation peak started immediately after contact of the roots with the cold bathing medium. Subsequently, the bathing medium was slowly warmed to room temperature (22 °C) without further external manipulation. Reproduced from ([124], p. 31), modified.

An extremely intriguing aspect of the phenomenon of root exudation is the sudden sharp pulses and oscillations in the rate that occurred at the scale of minutes for some (not all) detached roots of maize seedlings ([19] with references therein for earlier publications for different plant species; [22]) and for roots detached from 40 to 60-day-old sunflower plants [31,32]. The more pronounced pulses were in the observations with maize roots at 23 °C (Figure 5A reproduced from: [22]). The total volume of the studied roots was estimated at about 100 mm × 1 mm^2^ × π/4 or 79 mm^3^ (=μL); the pulses of 0.16 μL which occurred within 30 s are then 0.2% of the total root volume (including the root central cylinder with xylem vessels) [22]. Larger pulses, up to 1.5 μL at 30 °C making up to 4% of total root volume (these roots were 5 cm long,) were registered with much lower temporal resolution of 20 min in the wider capillaries for root segments of similar maize seedlings [26]. Converting the volume flow in the larger pulses over 20 min to 30 s will lead, however, to only 0.1% of total root volume/30 s (also Q_10_ of exudation at 30 °C compared to 20 °C was 3.2–3.5 [23] proportionally reducing the estimated pulses at 23 °C). Experiments with sunflower roots demonstrated exudation rates about 5–10 μL/minute and oscillations about 1–2 μL/minute for dry roots in air (exuding at the expenses of own intrinsic water) and for roots in tap water [31,32]. Based on the mentioned losses of weight by the roots (the roots were over 10 g), so the pulses were below 0.1%/30 s.

Oscillations of water uptake and exudation have been recorded for detached roots of 20–40-day old sunflower plants. The oscillations were in counterphase, were stimulated by 5 × 10^−5^ M acetylcholine and inhibited by 5 μM cytochalasin B, 10 mM 2,3-butanedione monoxime (potential inhibitor of nonmuscle myosin) and 1 μM CCCP (uncoupler of oxidative phosphorylation) [32]. Though the effect of inhibitors and stimulators could be nonspecific and related to the metabolic and structural components of cellular machinery, the counterphase of oscillations in water uptake and exudation may point to two distinct processes. Similarly, for excised onion roots and root segments directions of changes for fluctuating water uptake and water exudation were often opposite over 2 h intervals; moreover, the ratio between volume absorbed to volume exuded ranged from 0.65 to 1.04 over the intervals for one set of experiments ([10] with references therein to the earlier studies on periodic oscillations in root exudation). Aperiodic or periodic pulses in exudation for over 3 h were sometimes recorded for exudation from detached roots and from both basal and apical ends of maize root segments at the available scale of measurements (20 min) [26]. It is surprising that the sensitivity of measurements was not the factor which determined whether researchers paid attention or not to pulses of exudation. Sabinin [82] concentrated his efforts on the osmotic phenomena surrounding exudation from maize roots and its formalization; he developed high resolution of measurements below 0.001 μL (1 nl) [82]. However, he did not report and even rejected oscillations in exudation rate (apart from long-term temperature-dependent ones) [82].

Interestingly, for large (over 2–3 weeks old) maize roots when exudation was observed from the whole upper cut root surface, not only from the xylem vessels [35], but there were also periodic oscillations in exudation for some roots. The oscillations had a peak to peak period of 1–1.5 h and reached 1 g/(hour×root) for the large roots (Figure 4 of [35]).

The fastest so far documented exudation pulses of single roots are 0.16 μL/30 s or 0.2% of root volume/30 s (Figure 5 from [22]). These pulses need several factors to be fulfilled with one or several potential mechanisms realized. Firstly, a high hydraulic conductivity L_p_ should ensure the possibility of fast water fluxes. The known measured root and cell hydraulic conductivities L_p_ are sufficient to satisfy the observed high water fluxes: L_p_ of the order of 10^−7^ m s^−1^ MPa^−1^ was measured in hydrostatic experiments (0.94 ± 0.6610^−7^ from 0.1210^−7^ to 2.810^−7^ in endosmotic experiments where the direction of water flow was from the medium into the xylem) and about 10 times lower in osmotic experiments with maize roots [18]. It means that difference of 1 bar in hydrostatic pressure across a root (cell turgor could be involved) would lead to the flux of 10^-7^ m^3^ m^−2^ s^−^ hence per root stele area (based on Figure 1 of [35]: 100 mm × π × 0.4 mm = 125 mm^2^) it is 1.25 × 10^−2^ μL/second or 0.375 μL/30 s. The value twice exceeds the measured exudation pulses of 0.16 μL within 30 s [22]. It seems, however, that L_p_ could be a limiting factor and also it is unlikely that the exudation pulses could occur at the expense of the osmotic forces only. Secondly, large sudden ion fluxes should appear within the short time of the exudation pulses if we assume that water and ion fluxes are tightly linked. One explanation could be the activation of large mechanosensitive channels (MSC), allowing for the passage of water and ions, or of activating smaller MSCs which in turn activate larger ion channels of another types of ion channels. This would explain the inhibitory effect of gadolinium on exudation because it is known as an inhibitor of MSCs (recent reviews on plant mechanosensitive ion channels include: [167,168]), and also of cytochalasin, which has been shown to decrease the activity of MSCs, probably by reducing their mechanosensitivity [167]. Another option is a potential sharp short-term activation of cation-chloride antiporters [112,113,114], but since they are not well studied in plants (see for an overview [169]), we do not know whether the option is possible at all and what the mechanisms could be if any. Both mechanisms could be combined. Thirdly, more mechanisms at the subcellular level are proposed that involve changes of turgor pressure but not ion fluxes:

(1) cytoskeleton-based turgor oscillations of root cells constituting the so called metabolic component of root pressure [19];
(2) reverse osmosis realized in the plasmodesmata of endodermis when cell turgor oscillations around 1 Hz frequency lead to reduction of solute osmotic pressure [110,111].

Turgor pressure changes of cortical cells may be required for the oscillation peaks of measured exudation. Elastic modulus ε has been measured for many (39) cortical cells of maize roots from 12 to 211 μm from the root surface [18]. Turgor pressure varied from 1 to 6.6 bars, average hydraulic conductivities L_p_ were 2.4 ± 2.0 × 10^−7^ (from 0.5 to 8.7 × 10^−7^) m × MPa^−1^ × s^−1^, while elastic modulus ε varied from 1.3 to 18.1 MPa depending on the cell turgor [18]. We assume the average ε as 10 MPa, the total volume of cortex as 67 μL (total volume of cortex−olume of stele is about 79 mm^3^ − 12 mm^3^ = 67 mm^3^) and suggest that turgor P decreases for all the cortical cells (their diameters were from 26 to 43 μm and lengths from 205 to 305 μm: [18]). Then the estimates give the required ΔP for the cortical cells:

ΔP = 10 MPa × 0.16 μL/67 μL = 0.024 MPa or about 0.25 bars.

The estimates look reasonable if rather low; it is around the pressure of activation (around 0.1 bars) for some mechanosensitive ion channels of plants (see, e.g., Figure 1 and Supplementary Figures S1 and S7 from: [170]). Several separated symplastic cell domains could be considered within the root cortex (as was demonstrated for lateral root organogenesis in *Arabidopsis*: [171]), then the numerical estimates give higher turgor pressure changes/different hydraulic conductivities for the observed exudation pulses. There is a question, posed in [22], of how the activity of the cells within a domain is coordinated; presumably plasmodesmatal connections may link turgor pressures of individual cortical cells within symplastic domains and coordinate their activity.

One more intriguing observation about synchronization of exudation is that a strong exudation pulse was observed for groups of 24 maize roots measured collectively when they were transferred from warm (22 °C) to cold (4 °C) water; the pulse started at the very moment of immersing (resolution of these measurements reached 1 s). Exudation increased 8 to 56 times, went then down to zero, and gradually increased again when the water warmed up slowly ([124], p.31)). The phenomenon was taken as evidence for a biphasic exudation mechanism (Step 1: filling of the symplast, Step 2, release into the xylem) [124]. The exudation pulses were weakened but not totally suppressed by incubating the roots for 2 h in 10 mM KCN plus 10 mM SHAM (Salicylhydroxamic acid, blocking KCN-insensitive electron transport). However, addition of 200 mM sorbitol (4.8 bars or somewhat less) to the bathing medium (water) inhibited exudation completely. Weaker exudation peaks could be triggered by transfer of maize roots from 22 °C to 10–18 °C water [124]. The observation is similar to the earlier results with roots of maize seedlings, which expressed a sharp peak of exudation and contraction of root cross-section on being transferred from 15 ° to 30 °C or from 20 ° to 35 °C. The pulses of exudation (and decrease of measured root cross-section) were inhibited by 2,4-dinitrophenol and by *p*-chloromercuriobenzoate (influencing SH-groups of enzymes) ([13,14,15,16] and references therein). The researchers ([13,14,15,16] and references therein for similar sharp pulses of exudation revealed for sunflower roots transferred from 12 ° to 27 °C) suggested that cell contractile elements are responsible for the effect. However, the pulses of exudation could be explained by synchronous change of turgor pressure in large domains of root cells. Higher temporal resolution (within seconds) of exudation pulses and oscillations and a better understanding of root symplastic domains and their synchronized activity are the next steps for deeper investigation of oscillations in exudation and the mechanisms of root exudation in general. It is worth mentioning that measurements of root pressure by applied equilibrium hydrostatic pressure [8,9,17,18,37,38,172] did not report any oscillations (though the predicted oscillations are low, and this method could change the root water pathways which were suggested in [34] by influencing mechanosensitive elements). Additionally, as far as we are aware, the measurements of turgor pressure in individual root cortical cells also did not demonstrate any oscillation of turgor pressure at the time scale of minutes or less (e.g., [18] for maize).

## 8. Effects of Chemical Agents on Exudation and Root Pressure

Numerous publications have explored effects of a wide spectrum of chemical compounds on the exudation and water transport parameters, especially for 4–7 weeks old tomato roots [102], detached roots of 5–7 days old maize seedlings and 20–60 days old sunflowers roots ([19] and references therein for earlier publications, [22,23,24,25,26,27,28,29,30,31,32,33]). Table 3 illustrates some examples of how different substances influence exudation of detached roots and root segments of maize seedlings (from [23,24,25,26]). Further parts of the Section 8.1, Section 8.2 and Section 8.3 briefly describe groups of compounds according to their known mode of action with an accent on the ion channel blockers (the other types of compounds are discussed more in: [19] and publications from the laboratory).

### 8.1. Inhibitors of Metabolism and Substances Influencing the Integrity of Cell Membranes and Cytoskeleton

Inhibitors of metabolism or uncouplers of oxidative phosphorylation in roots (KCN, 10^−4^–10^−3^ M; 2,4-dinitrophenol, 1–5 × 10^−4^ M; carbonyl cyanide-3-chlorophenylhydrazone (CCCP), 10^−6^ M) have a clear inhibiting effects on exudation of sunflower and maize roots ([19,102] and references therein for earlier publications, [23,24,25,27,28,30,31,32]).

At the same time, inhibitors or destabilizing agents for intracellular contractile systems and cytoskeleton (*d*-tubocurarine, 6.7 × 10^−5^–1.5 × 10^−4^ M; cytochalasin B, 1–5 × 10^−6^ M; colchicine, 10^−3^ M; 2,3-butanedione monoxime, 1–10 mM; latrunculin B, 10^−6^ M) also inhibited exudation of maize and sunflower roots ([19] and references therein for earlier publications, [23,24,25,27,28,30,31,32]).

Substances that influenced the stability of cell membranes had effects on exudation, depending on their effects on membranes stability: compounds decreasing stability of cell membranes (pipolphen, 0.1–1 mM; EDTA, 7 mM) inhibited exudation for maize and sunflower roots while CaCl_2_ (1 mM) increasing stability of cell membranes stimulated exudation ([19] and references therein for earlier publications, [27,28,31,32]).

The effects clearly demonstrate that the living cells and their intracellular contractile and membrane systems are essential for root exudation and root pressure. Evidently, water and ion fluxes require the functioning of cellular water and ion membrane transport systems which are supported by ongoing cellular metabolism and by membrane interactions with the cytoplasmic cytoskeleton and intracellular compartments. From another point of view, the effects of relatively nonspecific compounds do not indicate the mechanisms of exudation. The application of the inhibitors at high concentrations for a long time could also disrupt root structure; for example, 2,4-dinitrophenol at 2.5 × 10^−4^ M resulted in the loss of turgor of maize seedling roots after 1–2 h of application [26].

### 8.2. Plant Hormones, Biologically Active Compounds, and Modifiers of Signal Transduction Chains

A stimulatory effect on exudation was discovered for plant hormones and structurally similar compounds: indole-3-acetyc acid, 10^−7^–10^−6^ M; kinetin, 10^−4^ M at 30 °C ([19] and the references therein for earlier publications, [28]) though kinetin at 10^−9^–10^−6^ M was reported to have inhibitory effect at 23 °C for similar roots of maize seedlings [173]. A stimulatory effect was shown for adenine, 10^−4^ M ([19] and the references therein for earlier publications); ABA, 10^−9^–10^−6^ M within first 5 h of application with further inhibition [173]; fusicoccin, 10^−9^–10^−6^ M within first 4–5 h of application with further inhibition [173]. The mechanisms of the phenomenon are not well understood, although some of the researchers ([19] and the references therein for earlier publications, [28] and further publications from the laboratory) suggest that the hormones enhance metabolism of the root cells which in turn results in exudation by activating nonosmotic component of root pressure (see below).

An interesting observation was that some animal neuromediators (acetylcholine, 10^−5^–10^−4^ M; adrenaline, 10^−6^–10^−5^ M; noradrenaline, 10^−5^ M; serotonin, 10^−4^ M) stimulated exudation ([19] and the references therein for earlier publications [24,25,28,30,31,32]). It led to questions about homology of regulatory systems in plants and animals at the cellular level. More specific and selective compounds, guanosine thiodiphosphate (2 × 10^–5^ M), a specific inhibitor of G-protein activation, and guanosine thiotriphosphate (2 × 10^–5^ M), a specific activator of G-proteins, also inhibited and stimulated, respectively, exudation of maize roots [33].

### 8.3. Ion Channel Blockers

Occasionally in the past, the influence of compounds, which later turned out to be ion channel blockers, were examined (see [15,174], and the references in the latter). The first intentional tests of ion channel blockers on exudation were reported [21,22]. Exudation from the cut surface of 24 or 30 maize roots was measured collectively with a micro drop recorder when agents were added to the bathing medium. A general problem with this approach is that it does not allow any statement about whether the agent actually reaches the membranes from which the measured exudate emerges. The most specific, consistent effect was found with gadolinium (Gd^3+^), which is a blocker of various MSCs [175], but also of (other) non-selective cation channels (see, e.g., [176]). At a concentration of 50 μM in distilled water as the bathing medium, exudation was increased initially, and then decreased down to 20% of the exudation of the control after 20 h [22]. The time lag between application and inhibition might be due to slow penetration of Gd^3+^ into the root (see [177]). The effect is hardly likely to be due to general cytotoxic effects (see [178]), but very recently an impact of rare earth elements like gadolinium and lanthanum at submillimolar concentrations on endoplasmic reticulum-plasma membrane contact sites has been shown [179]. In screening experiments with other potential blockers, La^3+^, which blocks a number of mechanosensitive ion channels, but not necessarily the same as Gd^3+^ [175], did not show any marked inhibitory effect on exudation at a concentration of 1 mM in the bathing medium [21,22]. This could be used as an argument against the involvement of an MSC, but also against explaining inhibition of exudation observed with gadolinium to less specific effects on membranes reported by [179]. In further screening experiments, the K^+^ channel blocker Ba^2+^, which permeates through Ca^2+^ channels, likewise increased exudation at first, and reduced exudation almost to zero within seven hours at a concentration of 3 mM in the bathing medium [21]. Application of a 3 mM solution of the K^+^ channel blocker quinine which permeates the lipid bilayer well resulted in a small and short increase of exudation followed within 2 h increase and a reduction of exudation to about a quarter of the initial level within 2 h and a further reduction to zero within the next 5 h. At a concentration of 100 μM quinine showed some reversible inhibitory effects [21]. Amiloride, inter alia a blocker of Na^+^ and of mechanosensitive cation channels (see [180]) showed some decrease of exudation at 100 μM concentration in the bathing medium, and complete exudation inhibition after 8 h at a concentration of 1 mM [21]; TEA^+^, a general blocker of K^+^ channels, penetrating the cell membrane very poorly, showed at 3–10 mM concentrations in the bathing medium an increase in exudation, but no following decrease [21]. Screenings with agents that interfere with chloride currents (NPPB, DPC, anthracene-9-carboxylic acid, sodium gluconate, and glutamate) showed inconclusive effects [21]. The results offer some support to the idea that xylem exudation strongly depends on potassium fluxes which are influenced by a mechanosensory system (see, e.g., [181]).

The initial xylem exudation by cationic channel blockers added to distilled water as a bathing medium poses a riddle. One might speculate that xylem exudation increases when rhizodermis exudation is blocked. But exudation also increased (up to double within 15–30 min) when submillimolar concentrations of NaCl and KCl were added to distilled water. Reversibility of the increase was tested for CsCl, LaCl_3_, KCl, and NaCl and confirmed for all these substances (and BaCl_2_, GdCl_3_, and TEA^+^), most markedly in the case of CsCl. When another salt was already present in the bathing medium the exudation increase weakened or failed to appear [22].

### 8.4. Summary on the Effects of Chemicals on Exudation

Though a large array of data has accumulated, it is difficult to decipher the mechanisms behind the inhibition or stimulation of exudation since most of the agents inhibiting and stimulating exudation (apart from probably fast effects of salts at low concentrations) have a complex influence on plant roots and their cells (including effects on metabolism, on membrane stability, intracellular signalling, even on gene expression and profiles of mRNAs, siRNAs, and transcriptional factors). However, generally speaking, the results provide solid support for a conclusion about the complexity of root pressure and exudation as it was described and conceived by Hales [1] and later on envisaged by further researchers at the developing levels of our understanding for biological systems (see, e.g., [19]). More specific inhibitors of ion channels support their role in driving ion fluxes linked to water fluxes. Cytoskeleton modifiers are suggested to directly influence cell turgor of root cells, although cytoskeleton elements are also tightly linked to regulation of ion channels.

## 9. Energy Activation Barrier and Osmotic Compensation Pressure for Root Exudation

The energy activation barrier for physico-chemical processes is also measured in physiology as Q_10_ (ratio of rates for a process when temperature changes by 10 °C) and often used to indicate the nature of reactions behind a process. Simple diffusion and osmosis have Q_10_ values close to 1 for temperatures 20 °C–30 °C (since ratio of 303 K/293 K in Δπ = C × R × T is close to one; the same for the simple diffusion equations). For more complex processes where temperature dependence is stronger and (simplifying the nature of processes) the form for the tail of Maxwell–Boltzmann distribution (developed for gases) is more shaped by temperature, Q_10_ reaches values of 2–3 and more.

For exudation of detached roots of sunflower and maize seedlings, the Q_10_ was around 2.5–4 for exudation rate measured at 30 °C divided by exudation rate at 20 °C (reviewed in ([19] with later references), [23,24,25,28,31,33]). However, the Q_10_ of root exudation for excised roots of maize dropped from over 3 to 1–1.3 under application of the inhibitors such as 2,4-dinitrophenol, cytochalasin B, colchicine [19], and to 1.7 from 3 after inhibition by guanosine thiodiphosphate with the background of decreased inhibited exudation rate [33]. On the other hand, stimulators of exudation such as CaCl_2_, indole-3-acetyc acid, acetylcholine, adrenalin, noradrenalin, and guanosine thiotriphosphate increased Q_10_ for excised roots of maize by 3–30% to 2.9–3.6 [19,28,33]. For twice faster exudation from the basal end of segments from the maize roots the Q_10_ was 3.7 ± 1.0 (same was for detached roots), but slower exudation from the apical end of segments had a statistically lower Q_10_ = 2.7 ± 1.2 [24]. The Q_10_ of exudation for surface-dried sunflower roots removed from water was surprisingly higher at 4.6 ± 0.7 compared to 3.6 ± 0.7 for roots in water [31].

Assuming that the mechanism of water transport/root exudation remains the same at the temperatures 20 °C and 30 °C, it is possible to use the simple Arrhenius equation [182] for the ratio of reaction rates *k*_2_*/k*_1_ and calculate the energy activation *E_a_* for root exudation:

*E_a_* = R ln(*k*_2_/*k*_1_)/(1/*T*_1_*−* 1/*T*_2_), (5)

where *T* is absolute temperature in K, R is the universal gas constant (8.31 J/(K×mole). For 30 °C and 20 °C and the high Q_10_ of exudation around 2.7 (e.g., Q_10_ = e) *E_a_* corresponds to 73.8 kJ/mole; for a Q_10_ of 5 the values are 1.6 times higher. In positioning this energy among biochemical and water transport parameters it is reasonable to compare the value of the activation barrier for water exudation with the other processes. For a biochemical reaction, 73.8 kJ/mole is about 2.5 moles ATP/mole of transported water (assuming standard free energy of ATP hydrolysis about −30 kJ/mole: see, e.g., [183] although it could be 1.5 less, down to −50 kJ/mole depending on the physiological conditions and concentrations). This estimate for direct active transport is not realistic, so the energy activation for the reaction comes from the other processes. Another comparison also looks unlikely, 73.8 kJ/mole being translated with sign reversion to water potential Ψ_w_ makes a huge −4350 MPa (−43,500 bars)—the value is over four times below the water potential of vapor with a humidity 0.1%, which probably does not exist on Earth. This high energy barrier of the reaction suggests that complex mechanisms participate in exudation and control the flows and water transport in roots although presumably the processes could be easily achieved by osmotic forces. Still it is not clear why and how the stimulators of exudation increase the energy barrier for exudation (increasing Q_10_ and, hence, energy activation for exudation).

**Figure 6:**
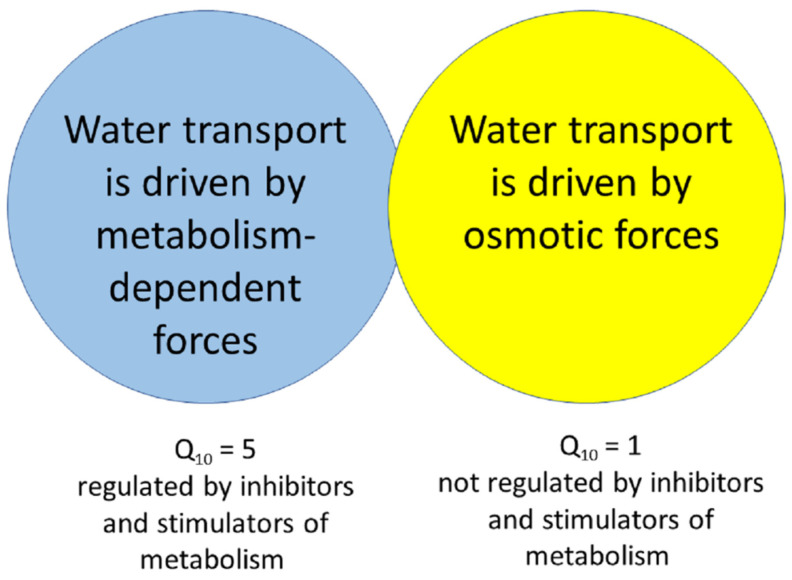
The generalized model of metabolic and osmotic components which constitute the root pressure, and are responsible for the observed exudation (based on: [19]). The separation between the components was based on an assumption that metabolism-dependent forces and processes are not of osmotic origin. The distinction between the components is supported by a complex of coinciding features (activation energy measured as Q_10_ increased parallel to exudation rate and compensation pressure to stop exudation when stimulators were applied; inhibitors decreased the three parameters). Oscillations in turgor pressure of root cells driven by the cytoskeleton (as suggested by in [19] to explain non-osmotic component) were neither supported nor rejected by sensitive single cell methods. The overlap between the circles demonstrates that both mechanisms operate together under most circumstances producing undistinguishable exudation flows.

Zholkevich [19] based on earlier results [14] developed the concept of two constituents of root pressure: an osmotic component with a simple physical nature and a Q_10_ around 1, and a so called metabolic component with a Q_10_ around 5 (Figure 6). The metabolic component is highly sensitive to inhibitors and bioregulators, the share of each component determines the rate of oscillations of exudation and the total Q_10_ of exudation [19]. The concept is highly valuable physiologically but still requires exact molecular entities and mechanisms to explain the existence of this metabolic component. A suggestion that cytoskeleton-linked microoscillations in turgor pressure of root cells create the water flow [19] has to be supported by sensitive techniques of cell and root pressure probes or by the methods of advanced imaging. One of the other limitations is that the concept of metabolic and osmotic components of root pressure ([19] and the references therein with further cited here) was developed in experiments with roots of maize seedlings and with sunflower roots only, so may not be applicable to the wider range of plant species including trees and vines.

The potential input and share of non-osmotic forces in root pressure and exudation is often estimated and measured by the osmotic compensatory method where an external solution with high osmotic pressure π_o_ is applied to stop exudation (Figure 2), this osmotic pressure π_o_ is taken as compensatory pressure. Then the osmotic pressure of the root exudate π_x_ is compared to the osmotic pressure of the external solution π_o_ and the part (if any) of external osmotic pressure exceeding the osmotic pressure of the exudate (π_o_–π_x_) is taken as the nonosmotic or metabolic component of root pressure (see, e.g., [19,102]). The experiments require sensitive methods to measure exudation and osmotic pressure and also need osmotically active compounds which do not permeate to the root tissues. So, mannitol used in early experiments is nowadays usually replaced by PEG with high molecular weight (e.g., 6000).

Contrary to purely osmotic concepts without nonosmotic forces (simple osmotic models from Table 2), experiments with tomato roots revealed that compensatory pressure is about 1.4 bars in distilled water with about 70% of a nonosmotic component while about 2.7 bars in nutrient solution with about 50% of nonosmotic component [102]. As the compensatory pressure was determined by mannitol solutions, root exudate was collected before the determination of the root compensatory pressure and it is possible that exudate became more concentrated when the exudation was stopped. However, the reversible twofold inhibition of root exudation and compensatory pressure by KCN, 10^−4^ M with lack of nonosmotic component was then in favor of a nonosmotic component [102]. Experiments with detached roots of sunflower demonstrated 40–70% of nonosmotic component for compensatory root pressure (compensatory pressure was equal to 1.6–2.2 bars by PEG) [19]. Another set of experiments with similar sunflower roots showed 40% of nonosmotic component, 1 bar of compensatory pressure with Q_10_ = 3.6 ± 0.7 for exudation (30 °C to 20 °C), while for the roots exuding without external water the nonosmotic component was 80% and Q_10_ = 4.6 ± 0.7 [31]. Similar experiments with roots and root segments of maize seedlings are in line with the results for sunflower roots. Briefly, the higher Q_10_ corresponded to a higher nonosmotic component of root pressure (measured by compensation method with external PEG 6000); stimulators of exudation increased both Q_10_ and the metabolic component of root pressure: lower exudation corresponded to lower Q_10_ and to the decreased metabolic component of root pressure [19,24,28,33]. Experiments with maize roots [173] demonstrated that exudation stopped at even 4.8–5 bars (not around three bars as in the abovementioned experiments by Zholkevich and colleagues) of external osmotic pressure by PEG 6000 and mannitol, respectively (close to the turgor pressure of the root cells). However, the researcher did not specifically compare the compensatory pressure to the exudate osmotic pressure [173].

**Figure 7:**
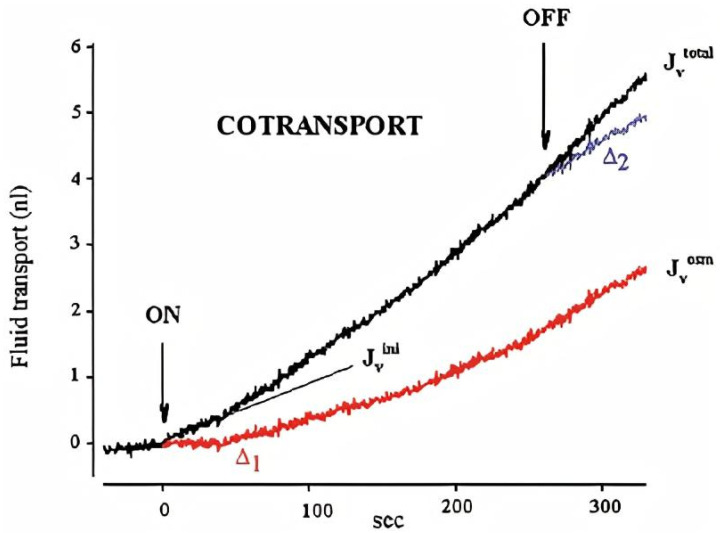
The two components of water transport by sodium–glucose cotransporter SGLT1. At point “ON” sugar was added to the external media, at point “OFF” the Na^+^–glucose cotransporter was turned off with a step jump of the membrane voltage to 0 mV. J_v_^total^ is the time course of the oocyte volume. There was an initial linear component J_v_^ini^ (continuous line, with slope 1.75 × 10^−3^ ± 3.85 × 10^−19^ cm s^−1^). Subtracting this component from the total J_v_ total yields the osmotic water flow J_v_^osm^. In the steady state, the slope was 4.41 × 10^−3^ ± 2.29 × 10^−5^ cm s^−1^. The osmotic water flow occurred after a 40 s delay (the slope in the steady state was 2.66 × 10^−3^ ± 2.29 × 10^−5^ cm s^−1^). Δ1 and Δ2 are the changes in slope of the fluid transport vs. time curve (i.e., the differences in the rates of fluid transport), which would be predicted if Na^+^–glucose cotransport was turned on and off). Reproduced from [184] with the permission of the publisher John Wiley and Sons with further enhancement of pdf by AI software.

Interestingly, with this approach at the molecular level a similar nonosmotic component was discovered and characterized under heterologous expression in oocytes for cotransport of water with substrates (sugars or cations) for specific animal transporters [116,117,118,119]. Co-transport added an extra component to the osmotic water flow, of nearly 50% (Figure 7 and Figure 2 reproduced from [184]). Similar transporters can function in plants; the transporters could be responsible for the energetically uphill (that is nonosmotic) water transport in roots [112,113,114]. Calculations for the possible energetically uphill water transport to the xylem were based on measured membrane potential of xylem parenchyma cells and hydraulic conductivity L_p_ of their plasma membranes. The conclusion was that for typical L_p_ around 10^−7^ m s^−1^ MPa^−1^ extra root pressure developed by the mechanism may exceed 1.2 bars reaching over 10 bars at 10 times lower (still well within the range of measurements, see above) L_p_ [112].

Indeed, simple calculations demonstrate that hydrolysis of one mole of ATP molecules (see above) releases 30–50 kJ, while for transport of 1 mole of water against pressure of 1 bar we need only (0.018 kg × 9.81 H/kg × 10 m) 1.77 Joules. So, hydrolysis of one molecule of ATP is energetically sufficient for transport of over 15,000 water molecules against 1 bar of osmotic pressure. For an exudation pulse of 0.16 μL/30 s it would correspond to change of around 6 × 10^−10^ moles of ATP per a root (equivalent to about 8 μM per a root of 80 mm^3^ while regulatory K_m_ for maize root H^+^-ATPase is over 10 times higher [185]). So, we believe that finally the research will lead to molecular identities and mechanisms of how metabolism of living cells is expressed in the measured root exudation.

Still, the experiments with osmotic compensatory pressure need more detailed analysis. Firstly, a check of osmotic pressure π_x_ for exudates under increasing external osmotic pressure π_o_ is required. Lower exudation rate may coincide with higher exudate osmotic pressure π_x_; hence, it would increase the part of osmotic component (higher π_x_ at lower exudation was indeed demonstrated by [102] but the interpretation was the opposite to that given here). Under low exudation rate the collection of exudates and measurements of their osmotic pressure π_x_ may require nanoliter sampling and nanoliter osmometry with further technical advances. Secondly, direct summation of Q_10_ for separate components of the process based on their percentage of the whole root compensatory pressure may not be valid and would require complex models. Thirdly, molecular and genetic manipulations with ion transporters and mechanosensitive ion channels could help to reveal whether they do participate in the formation of root pressure and its metabolic component. Last, but not least, the experiments with wider range of plants are required to understand the role of the nonosmotic water transport for different groups of plants.

## 10. Proposed Sketch of the New Mechanism Which Explains Both Xylem and Rhizodermal Types of Exudation; Overall Conclusions and Further Perspectives

Our overview describes how the phenomena of root exudation and root pressure were studied over centuries and how the knowledge in the direction accumulated and interconnected with ideas from the adjacent areas, how it further developed in models being paralleled by experimental advances. Pfeffer [87,91] had already suggested that there might be several mechanisms of root exudation. We also tend to conclude that there are several mechanisms involved in exudation that likely operate under specific conditions for different plant species. Possible mechanisms start with simple osmosis, which is an inevitable consequence of the basic laws of physical chemistry and the likely origin of, for example, stem and xylem pressure for sugar maple [164,165] and for walnut [166]. Other mechanisms employed by more complex models foresee a direct contribution of living cells to exudation involving several other processes: from reverse osmosis at the level of the symplast (e.g., [106]) or at the subcellular level [110,111] to co-transport of water with ions via cation-chloride or other cotransporters [112,113,114] similar to suggested formation of cerebrospinal fluid in mammals [186]. Here, we paid particular attention to a mechanism with proposed turgor oscillations of root cells due to functioning of cellular cytoskeletal elements ([19] with references therein and further works from the laboratory) and outlined the mechanism whereby activation of mechanosensitive ion channels is suggested as a basis for periodic changes in reflection coefficients of hydraulically isolated domains in roots or compartments, rhizodermis exudation, and pulses of exudation [22]. When we observe the total summed water and ion fluxes, the role and participation of each mechanism often remains obscure and needs further investigation.

**Figure 8:**
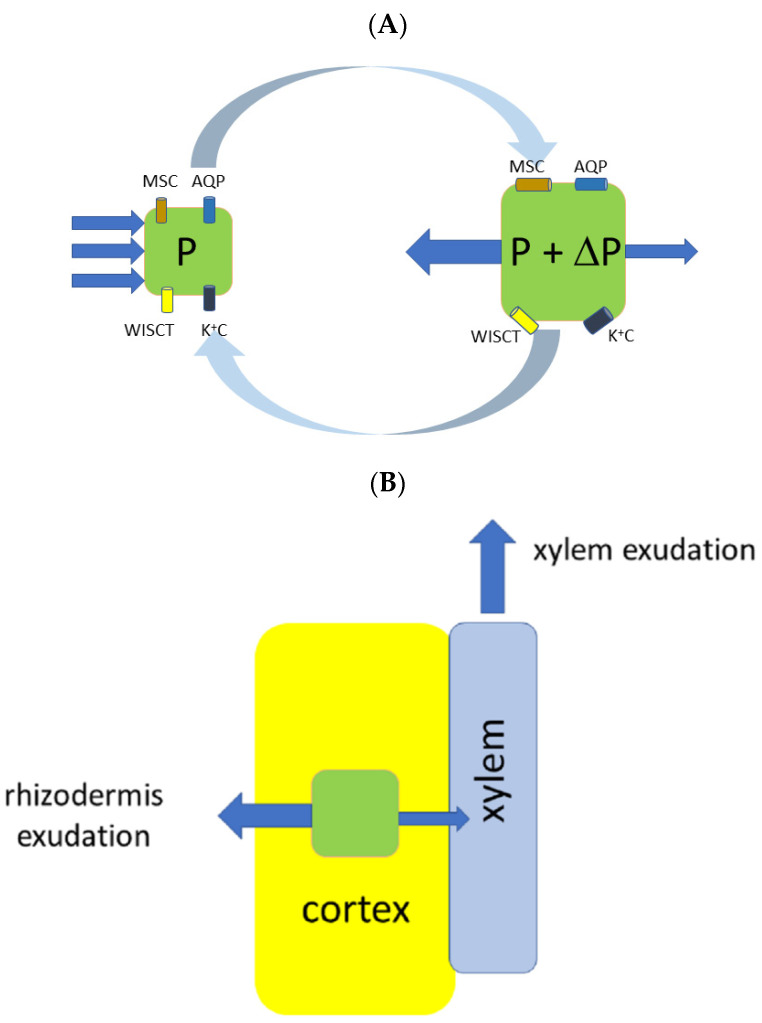
Scheme demonstrating how a domain of hydraulically linked cells in the root cortex potentially generates both xylem and rhizodermis exudation. (**A**). Suggested model for hydraulically linked cells forming a domain with alternating turgor pressure P and water fluxes (blue arrows). The cells within the domain increase their turgor P to the certain threshold of turgor pressure P + ΔP (active water transport could be involved via water-ion/substrate coupling transporters (WISCT) including known cation chloride cotransporters and cation substrate cotransporters), then mechanosensitive ion channels (MCS) activate the opening of other ion channels (e.g., potassium channels K^+^C with depolarization of membrane potential and outward K^+^ fluxes) or activation of aquaporins (AQP) generating pulses of water flows. The process repeats, the sizes are disproportionally enlarged with more details in the text. (**B**). The resulting water flows from the domain of hydraulically linked cells (green box) produce both strong pulses of xylem exudation and rhizodermis exudation. More details are in the text. It should be noted that not all the roots exhibited exudation pulses, so the scheme is limited to certain roots only.

Examination of the origin of the plant root liquid exudates showed that they arose at first from all kinds of living cells and only later from open xylem vessels. This observation suggests that at least one type of exudation is caused by the osmoregulation in living cells. Such an explanation would very neatly fit the observation of concomitant rhizodermis and xylem exudation. The observations of Cailloux [132,133] indicate that there might be an individual, independent exudation, and absorption behaviour of single root cells. If the osmoregulation of single cells is synchronized, strong exudation pulses can occur. Still the model of Schwenke and Wagner [22] seems promising in explaining these phenomena. It postulates the involvement of mechanosensitive channels in cellular osmoregulation in order to explain the observed effects of gadolinium and cytoskeleton inhibitors on exudation and also the explosiveness of some exudation pulses. When a threshold turgor is reached, mechanosensitive ion channels open; ions are released from the cell either through the MSC themselves or through other channels triggered by the opening of these MSC. Water may follow osmotically [22] or could be also co-transported with ions through membrane channels [112,113,114] (Figure 8A). Because no standing gradients in maize root vessels were found [108,109], re-uptake of ions from the vessels should be assumed by the model. Uphill water transport could occur through cation channels where the water-ion coupling ratio is larger for ion efflux than for ion reuptake (see for such a concept in ([112], p. 386, Figure 2). Uphill transport could also be achieved by a higher channel density at the vessel sites than at the root surface (see [22]). The model would also fit nicely observations of a phase shifting of root water uptake and root xylem exudation (see [19] and the references therein for earlier publications; [32]).

Here, we introduce a simple sketch for the model at the level of roots (Figure 8B). The model tries also to explain both xylem and rhizodermis exudation with presumed specific features for each type of exudation. The suggestion is that the metabolically-active component of water transport (due to active ion fluxes, turgor oscillations, or activity of the specific transporters) participates in both xylem and rhizodermis types of exudation. Upon reaching a certain threshold of turgor, the mechanosensitive ion channels of cells are activated, which results in the release of ions followed by water (Figure 8A). Most likely opening the channels leads to changes in potassium permeability since the ion is among the main osmolytes and far from thermodynamic equilibrium controlled by membrane potential of the cells. So, for example, depolarization of cells would release plenty of K^+^ (based on Nernst equation) followed by outward water fluxes. We further ignore, for the sake of extreme simplicity, the complex radial root structure and assume homogeneous hydraulic conductivity in both directions but do take into account small volume of xylem compartment with high hydraulic resistance along the xylem vessels and between them; we also assume an unlimited volume of outer compartment with negligible hydraulic resistance (similar to idea of different compartments from the reverse osmotic exudation model: [106]). Hence, any released water would flow both directions, to the xylem and to the outside of the root (Figure 8B). In this way both xylem exudation and rhizodermis exudation would occur with expected pulses in exudation. Presumably, based on the physical assumptions, more water with lower concentration of ions would be released via rhizodermis exudation while less water with higher concentration of ions and higher pulses in exudation would be released via xylem exudation, though the assumption is rather arbitrary and needs calculations with further experimental verification for proof.

In reality, the complex radial root structure, differences in hydraulic conductivities and other numerous factors should be taken into account to compose a detailed picture how concerted activity of cell domains produces observed exudation peculiarities. The existence of domains with higher turgor pressure would not necessarily require the activity of water-ion cotransporters, the water-ion cotransporters could add extra turgor pressure and their role has to be estimated and evaluated separately. The framework of this short review does not allow us to develop here the proposed model in detail. We are at the very beginning of the road since we do not know about the regulation and the properties of the transporters and of the mechanosensitive channels related to water transport: we do not even know which ones are involved in the proposed scheme.

Further study of root exudation would benefit from the new opportunities from genetics and molecular biology with mutants in specific genes, CRISPR editing, and single point mutations in the genes of mechanosensitive ion channels and water-ion/substrate cotransporters which were proposed as molecular candidates for conversion of ATP-driven ion fluxes or turgor changes to directed water fluxes. Still, however, we are at the very beginning of identifying the molecular entities for the processes. At the same time, biophysical experiments combining exudation studies with root pressure probe, cell pressure probe, picoliter osmometry, and with measurements of membrane potential of cells in exuding roots should provide more information about mechanisms of root exudation, a phenomenon which seems an unresolved puzzle yet.

## Figures and Tables

**Table 1:** Osmotic pressure (II_i_), pH values, concentrations of K^+^, Ca^2+^, Cl^-^ (mM), free amino acids (mM), sugars (μg/0.1 mL) in exudate from detached roots of *Zea mays* L. seedlings.

	II_i_, MPa	K^+^	Ca^2+^	Cl^-^	Amino Acids	Sucrose	Fructose	pH
Exudation from detached roots	0.13 ± 0.03	17.2 ± 2.9	2.4 ± 0.8	18.7 ± 3.2	7.8 ± 3.5	6 ± 1	29 ± 11	5.2 ± 0.2

Data are given as means ± SD. Exudate was collected and united for thirty-sixty detached roots (apical 5 cm of roots of 5–7 days old seedlings) after 1 h of exudation at 30 °C and frozen if stored before analyses. Based on the results from [23,24].

**Table 3:** Effect of 2, 4-dinitrophenol, various inhibitors of contractile proteins and biomediators on exudation rate (J_w_) from detached roots and root segments of *Zea mays* at 30 °C over the first 20 min of the process.

Treatment	J_w_, μL × cm^−2^ × h^−1^		
	Detached Roots	Segments	
		Basal End	Apical End
No treatment (water)	1.9 ± 1.0 (100)	1.6 ± 1.2 (100)	0.7 ± 0.5 (100)
2, 4-Dinitrophenol, 2.5 × 10^−4^ M	0.5 ± 0.4 (26)	0.4 ± 0.4 (25)	0.2 ± 0.3 (29)
Cytochalasin B, 2 × l0^−5^ M	1.3 ± 1.0 (68)	1.2 ± 1.2 (75)	0.5 ± 0.3 (71)
*d*-Tubocurarine, 6.7 × 10^−5^ M	1.1 ± 0.5 (58)	0.9 ± 0.8 (56)	0.5 ± 0.3 (71)
Acetylcholine, 10^−4^ M	2.2 ± 0.9 (116)	1.7 ± 0.7 (106)	1.1 ± 0.8 (157)
Noradrenaline, 10^−5^ M	2.3 ± 1.1 (121)	2.1 ± 1.2 (131)	1.2 ± 1.0 (171)

Data are given as means ± SD. 95% confidence intervals are at least 3 times less than SD. Percent of the corresponding control values is given in the parenthesis. The differences from control treatments are significant at *p* = 0.95 level; stimulation of exudation from the apical end by biomediators significantly differs from other root segments at *p* = 0.95 level. Stimulating effect of acetylcholine and noradrenaline increased with time (2nd hour of exudation) for exudation from detached roots and apical end of segments (from: [23,24,25,26]).

## Data Availability

Available data are presented in the manuscript.
